# 

**Published:** 1884-10

**Authors:** 


### ANNOUNCEMENT.

---

*To the Public:*

The undersigned having sold his entire interest in and control of *The Atlanta Medical Register*, (formerly *The Atlanta Medical and Surgical Journal,*) to Messrs. James P. Harrison & Co., takes pleasure in commending THE JOURNAL under its new proprietorship to the Medical Fraternity and the public generally.

Messrs. James P. Harrison & Co. have splendid facilities for publishing THE JOURNAL in first-class style, and have determined to enlarge and improve it so that it shall be eminently worthy and valuable to the Medical Fraternity.

The new volume will commence with the March number. Those who have paid their subscriptions in advance to me will be supplied by the new proprietors until the end of the term for which they have paid. Those who are in arrears will please, in renewing, include in the sum they may remit, the amount due to February, 1884, for which Messrs. James P. Harrison & Co. will account to me.

H. H. DICKSON, PUBLISHER.

---

It will be seen by the above announcement that *The Atlanta Medical Register* (formerly *The Atlanta Medical and Surgical Journal,*) heretofore owned and published by H. H. Dickson, has been purchased by the undersigned, and will hereafter be issued under the old name,

### THE ATLANTA MEDICAL AND SURGICAL JOURNAL.

It will be enlarged to SIXTY-FOUR pages, and will be printed in first-class style, on good paper, from clear new type.

THE JOURNAL will be under the editorial management of W. F. Westmoreland, M.D., Professor of Surgery in the Atlanta Medical College; H. V. M. Miller, M.D., Professor of Practice of Medicine in the Atlanta Medical College, and James A. Gray, M.D., Lecturer on Venereal Diseases, Atlanta Medical College. They will be assisted by a competent corps of special contributors.

A special feature of THE JOURNAL will be the illustration of all matter that requires illustrating. These illustrations will be furnished by the Publishers free of charge to contributors.

It will be the desire of the Publishers to furnish a Journal first-class in every respect, and they solicit the profession to sustain them in their efforts.

The time and expense necessary upon transferring THE JOUR[illegible]L, changing the form, type, etc., will preclude the possibility of issuing a February number. The f[illegible] number of the new series will appear about the 15th of March, and regularly thereafter on the 1[illegible]f each month.

The subscription price of the new JOURNAL has been fixed at $2.50 per annum. Subscribers to *The Register* will be furnished with the new JOURNAL until the expiration of their subscription.

*JAS. P. HARRISON & CO., Publishers,*

*Atlanta, Georgia.*

Do us the favor when sending your own subscription, to induce as many others to join you as possible.

☞ Single subscribers, $2.50 per annum; clubs of five, $2.00 per annum.

ing from the requisites for understanding the matter treated of, and in these days of book-making, it is a most commendable result to get up so small a volume upon such an important subject, which covers the ground to the entire satisfaction of the searcher after knowledge in this department.

It is a labor-saving process for the student, and is entitled to the careful study of all concerned.

---

### BOOKS AND PAMPHLETS RECEIVED.

Henke's Atlas of Surgical Anatomy. A series of plates illustrating the application of anatomy to medicine and surgery. Translated and edited by W. A. Rothacker, M.D., Pathologist to Cincinnati Hospital, and Lecturer on Pathological Anatomy in Miami Medical College. Cincinnati, A. E. Wilde & Co. 1884.

A Manual of Diseases of the Throat and Nose, including the Pharynx, Larynx, Trachea, Œsophagus, Nose and Naso Pharynx, by Morell Mackenzie, M. D., London, Consulting Physician to the Hospital for Diseases of the Throat; Lecturer on Diseases of the Throat at the London Hospital, Medical College, and Corresponding Member of the Imperial Royal Society of Physicians of Vienna. Vol. II.—Diseases of the Œsophagus, Nose and Naso Pharynx. New York, Wm. Wood & Co. 1884.

Visions of Fancy. A Poetical Work, by N. M. Bassett, M. D., St. Louis, Mo. Commercial Printing Company, 4 & 5 N. Third St. 1844.

Life and Public Services of Grover Cleveland, by Pendleton King. New York and London G. P. Putnam's Sons. 1884.

Medical Communications of the Massachusetts Medical Society. Vol. XIII, No. 3. 1884.

The Care and Feeding of Infants. Boston. Doliber, Goodale & Co. 1884.

Public Health Laws of Illinois and Sanitary Memoranda. Springfield. Illinois State Board of Health. 1884.

Removal of a Piece of Iron from the Vitreous Chamber, by means of the Magnetic Needle. By Julian J. Chisholm, M. D., Professor of Eye and Ear Diseases in the University of Maryland, Surgeon in charge of the Presbyterian Eye, Ear and Throat Charity Hospital, etc., etc. Reprint from Transactions of the Medical and Chirurgical Faculty of Maryland. 1884.

Hyperæsthesia. A paper read before the Medical Association of the State of Alabama at its session held in Selma, April, 1884. By James T. Searcy, M.D., Tuscaloosa, Alabama, 1884. Reprint from the volume of Transactions.

The Influence of Lung Retractility in Pleurisy and Pneumo-Thorax. By F. Donaldson, M.D., Clinical Professor of Diseases of Throat and Chest of the University of Maryland; Ex-president of the Medical and Chirurgical Faculty of Maryland; Fellow of the American Laryngological Association; Fellow of the American Climatological Association, etc., etc. Reprint from Transactions of the Medical and Chirurgical Faculty of Maryland, 1884.

Annual Announcements—1884-5, Medical Colleges: National University, Washington, D. C.; The College of Physicians and Surgeons, Boston; Medical College of Alabama, Mobile; Woman's Medical College, of Baltimore.

Resolved, That the sincere sympathy of this Board be most respectfully tendered the sadly stricken family, and that a page in our records be inscribed to his memory.

Resolved, That these resolutions be published in the city papers, in the ATLANTA MEDICAL AND SURGICAL JOURNAL and in the Southern Medical Record.

W. S. ARMSTRONG, M. D.,
*President.*
JAMES B. BAIRD, M. D.
*Secretary.*
FRANK P. RICE,
AARON HAAS.

### PUBLISHERS' NOTICE.

There are a number of our subscribers who are due THE JOURNAL for subscriptions, some for a longer time than one year. We will in the next few days send bills to every one, and will expect them to remit at once. We are giving you a first class journal in every respect, and must have the money to keep it up to the present standard.

JAS. P. HARRISON & Co.
*Publishers.*

### OUR ADVERTISERS.

MESSRS. BATTLE & Co., ST. LOUIS, have a new advertisement in the JOURNAL this month. Of one of their preparations, C. A. Bryce, editor *Southern Clinic*, Richmond, Va., says: "I have had the most gratifying experience with Iodia in syphilis. I have treated many hundreds of cases with it, and it is decidedly the best preparation I have ever used for constitutional syphilis, after the moderate use of mercury. Indeed, I generally use it in all cases of syphilis in the final treatment. If I had to select one combination of agents for the entire treatment of constitutional syphilis, after bringing the system under mercurial influence, it would be Iodia."

THE GATE CITY STONE FILTER.—Elsewhere in this issue will be found an advertisement of this filter, manufactured and sold by McBride & Co., of this city. The great objection to most filters is that they soon become saturated with matter extracted and become a source of pollution themselves.

This objection cannot be raised against the "Gate City Stone Filter," because the filtering and purifying is complete upon the surface of the filtering medium, it being impossible for any matter to penetrate because of its close porousness; this feature is of the greatest value, for to clean it the accumulation can be simply brushed and rinsed from the surface, and the Stone Filter is as good as new.

We have used this filter and unhesitatingly recommend it.

A. A. MELLIER, ST. LOUIS.—This gentleman is sole proprietor and manufacturer of Tongaline. We extract the following notice of the preparation from the St. Louis *Brief* of recent date:

"In those forms of neuralgia and rheumatism of a malarial origin, and most seem to be such, I have been highly gratified by the action of TONGALINE in conjunction with quinine, the therapeutic properties of both seeming to be accentuated under these circumstances.

"With each dose of TONGALINE I prescribe two to five grains of quinine, according to the severity of the case and the susceptibility of the patient to the effect of the latter.

"Thus far, have not experienced a single failure."

WARREN'S FOOD FLOUR.—This celebrated all-wheat flour is now on sale in this city by Mr. Volney Dunning, corner of Broad and Marietta streets. We feel sure that parties trying it will be pleased. See the new advertisement of it to be found elsewhere in THE JOURNAL.

GOODWYN & SMALL, MACON, GA.—These gentlemen are now manufacturing on an extensive scale a compound syrup of hypophosphites with lactates and pepsin. Elsewhere in this issue will be found their advertisement in which they publish the formula. From our knowledge of the preparation we have no hesitancy in recommending it to the favorable consideration of the profession.

MAGNUS & HIGHTOWER, ATLANTA.—Attention is directed to an announcement elsewhere by this firm. The gentlemen composing the firm are entirely trustworthy. Physicians or others ordering drugs, chemicals, instruments, etc., from them can depend upon getting just what they order, and at prices as low as the same goods can be sold in New York or Philadelphia. They have a large line of dissecting cases to which the attention of students is directed.

SHARP BROS., ATLANTA.—We take pleasure in calling attention to the advertisement of Messrs. Sharp Bros. at 202 Marietta street. They keep a full line of the purest and best drugs; their prescription department is supplied with Squibb's goods exclusively. Physicians in the city may feel assured that their prescriptions will be accurately filled by a competent prescriptionist, if they are sent to this place.

### CONTENTS.

ORIGINAL COMMUNICATIONS—

### NOTICE.

Original communications solicited. When articles require illustration the engravings will be furnished by the publishers free of charge.

Subscription: $2.50 a year.

Subscribers will please not send us money by Postal Notes. Remit by Express, Draft, Post-office Order, or Registered Letter.

Address all letters and books, and make all drafts payable to

The Atlanta Medical and Surgical Journal,
Care Jas. P. Harrison & Co., Atlanta, Ga.

